# ABCA8-mediated efflux of taurocholic acid contributes to gemcitabine insensitivity in human pancreatic cancer via the S1PR2-ERK pathway

**DOI:** 10.1038/s41420-020-00390-z

**Published:** 2021-01-11

**Authors:** Chunmei Yang, Hui Yuan, Jinyang Gu, Dengfei Xu, Mingwei Wang, Jie Qiao, Xi Yang, Jian Zhang, Ming Yao, Jianren Gu, Hong Tu, Yu Gan

**Affiliations:** 1 State Key Laboratory of Oncogenes and Related Genes, Shanghai Cancer Institute, Renji Hospital, Shanghai Jiao Tong University School of Medicine, Shanghai, China; 2 Department of Thoracic Surgery, Cancer Research Center, Fudan University Shanghai Cancer Center, Shanghai, China; 3 Department of Oncology, Shanghai Medical College, Fudan University, Shanghai, China; 4 Department of Transplantation, Xinhua Hospital, Shanghai Jiao Tong University School of Medicine, Shanghai, China; 5 Department of Oncology, Henan Province People’s Hospital, Zhengzhou, Henan Province China; 6 Department of Radiation Oncology, The Third Hospital Affiliated to Nantong University, Nantong, Jiangsu Province China; 7 Department of Radiation Oncology, Fudan University Shanghai Cancer Center, Shanghai, China; 8 Department of Medical Oncology, Fudan University Shanghai Cancer Center, Shanghai, China

**Keywords:** Pancreatic cancer, Cancer therapeutic resistance

## Abstract

The development of resistance to anticancer drugs is believed to cause chemotherapy failure in pancreatic cancer (PC). The efflux of anticancer drugs mediated by ATP-binding cassette (ABC) transporters is a widely accepted mechanism for chemoresistance, but for ABCA subfamily members, which are characterized by their ability to transport lipids and cholesterol, its role in chemoresistance remains unknown. Here we found that the expression of ABCA8, a member of ABCA subfamily transporters, was significantly increased in human PC cells after gemcitabine (GEM) treatment, as well as in established GEM-resistant (Gem-R) PC cells. Importantly, ABCA8 knockdown reversed the chemoresistance phenotype of Gem-R cells, whereas ABCA8 overexpression significantly decreased the sensitivity of human PC cells to GEM, both in vitro and in vivo, demonstrating an important role of ABCA8 in regulating chemosensitivity. Moreover, our results showed that treatment with taurocholic acid (TCA), an endogenous substrate of ABCA8, also induced GEM insensitivity in PC cells. We further demonstrated that ABCA8 mediates the efflux of TCA out of PC cells, and that extracellular TCA activates extracellular signal-regulated kinase (ERK) signaling via the sphingosine 1-phosphate receptor 2 (S1PR2), which is responsible for ABCA8-induced GEM ineffectiveness. Together, these findings reveal a novel TCA-related mechanism of ABCA subfamily transporter-mediated chemoresistance that goes beyond the role of a drug pump and suggest ABCA8 or the TCA-S1RP2-ERK pathway as potential targets for improving the effectiveness of and overcoming the resistance to chemotherapy in PC.

## Introduction

Pancreatic cancer (PC) is the fourth leading cause of cancer-related death with a 5-year relative survival rate of only 9% and its incidence rate has persistently increased in past decades^1,2^. Although surgery is the current standard of care for resectable PC, chemotherapy is essential for PC treatment either in an adjuvant setting after surgical resection or in the case of unresectable advanced disease^3^. However, the emergence of chemoresistance limits treatment efficacy and inevitably translates into poor clinical outcomes^4,5^. Therefore, clarifying the molecular mechanisms underlying the chemoresistance of PC is an urgent need.

ATP-binding cassette (ABC) transporters are known to play a pivotal role in the development of PC chemoresistance due to their ability to pump anticancer drugs out of cancer cells^6^. More than 40 ABC transporters have been identified in human and are divided into 7 subfamilies (ABCA–ABCG) based on amino acid sequence similarities and phylogeny^7^. The most extensively studied ABC transporters related to chemoresistance include ABCB1 (also named P-glycoprotein or multidrug resistant gene 1), ABCC1 (also named multidrug resistance protein 1), and ABCG2 (also named breast cancer resistance protein)^8^. These ABC transporters have been demonstrated to confer PC resistance to gemcitabine (GEM)^9–11^, which is the mainstay chemotherapeutic agent for PC. Several other members of ABCB or ABCC subfamily, such as ABCB2^12^ and ABCC5^13^, have also been found to be associated with PC chemoresistance. However, relatively little is known about the roles of ABCA subfamily transporters in drug resistance.

The ABCA subfamily of transporters consists of 12 members. It was initially recognized for its physiological functions related to transport lipids and cholesterol^14^. For example, ABCA1, ABCA5, and ABCA7 actively export cholesterol and phospholipids^15–18^, and ABCA8 stimulates cholesterol and taurocholic acid (TCA) efflux^19^. A number of previous studies focusing on ABCA transporters reinforced their roles in the maintenance of lipid homeostasis and disorders related to lipid transport^14^. Recently, the effects of ABCA transporters on tumor progression have attracted increasing interest. Regarding their roles in chemotherapy, the expression of several ABCA transporters, such as ABCA1 and ABCA12, has been reported to be associated with a reduced response to paclitaxel/FEC (5-fluorouracil, epirubicin, and cyclophosphamide) neoadjuvant chemotherapy in breast cancer patients^20^. ABCA3 expression defined a class of cancer stem-like cells with high chemoresistance^21^. However, their functional roles in regulating chemo-responsiveness necessitate detailed investigations.

Our recent study found that enriched housing environment-stimulated eustress or positive psycho-social stress increased the sensitivity of mouse PC to GEM and 5-fluorouracil and indicated the involvement of the murine ortholog of human ABCA8 in the regulation of chemosensitivity by eustress^22^. Only a few studies have linked ABCA8 to chemosensitivity in human cancers. A gene expression screen identified a marked increase in ABCA8 expression in the paclitaxel-resistant variants of ovarian cancer cells^23^. In addition, high ABCA8 expression was associated with poor outcome in ovarian cancer^24,25^. However, the functional roles of ABCA8 and its underlying mechanisms in regulating the chemosensitivity of human cancer remain unknown.

Using both in vitro and in vivo models of human PC, we provide evidence demonstrating that ABCA8 is involved in the chemoresistance of PC cells, and that ABCA8 overexpression leads to a significant decrease in sensitivity to the anticancer drug GEM. Moreover, we further characterized a novel TCA-related mechanism by which ABCA8 induced GEM ineffectiveness beyond acting as a drug pump in PC cells.

## Results

### ABCA8 is upregulated in GEM-treated PC cells and in GEM-resistant (GEM-R) PC cells

**Fig. 1:**
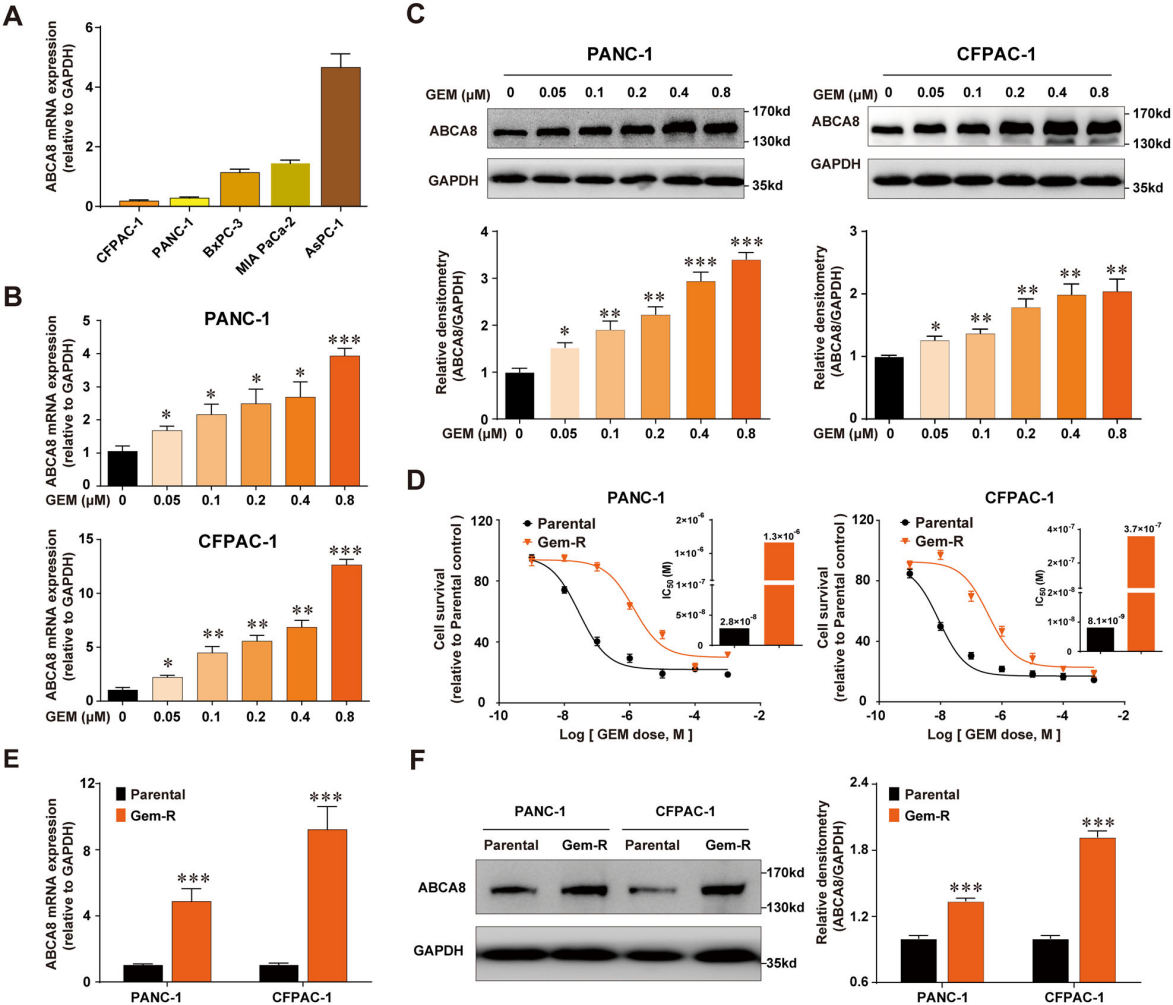
ABCA8 is upregulated after GEM simulation and in Gem-R cells. **A** ABCA8 mRNA expression in various PC cells was measured by quantitative real-time PCR (*n* = 3 independent biological repeats). **B**, **C** PANC-1 and CFPAC-1 cells were treated with GEM at the indicated doses for 72 h, and ABCA8 mRNA and protein levels were measured by quantitative real-time PCR (**B**) and western blotting (**C**). *n* = 3 independent biological repeats for quantitative real-time PCR or western blot analyses. The histograms in **C** show the densitometric analyses of the bands. **D** Relative sensitivity of Gem-R pancreatic cancer cells compared with parental cells was determined by CCK-8 cytotoxicity assays (*n* = 4 independent biological repeats). Histograms show the comparison of IC_50_ values between Gem-R cells and parental cells. **E**, **F** Relative ABCA8 expression in Gem-R and parental cells was determined by quantitative real-time PCR (**E**) and western blotting (**F**). *n* = 3 independent biological repeats for quantitative real-time PCR or western blot analyses. The histogram in **F** shows the densitometric analysis of the bands. *P < 0.05, **P < 0.01, and ***P < 0.001.

We first examined the changes in ABCA8 expression in response to GEM stimulation in PANC-1 and CFPAC-1 cells, which had relatively low intrinsic expression of ABCA8 among the five human PC cell lines we tested (Fig. 1A). As shown in Fig. 1B, C, ABCA8 expression was significantly increased by GEM treatment in a dose-dependent manner at both the mRNA and protein levels. Next, we assessed whether ABCA8 was persistently expressed at a high level in Gem-R cells. Gem-R PC cell lines were established by exposing PANC-1 and CFPAC-1 cells with stepwise increasing concentrations of GEM for 4 months. The half-maximal inhibitory concentration (IC_50_) values of Gem-R cells showed that the cells were ~50-fold more resistant to GEM than parental cells (Fig. 1D). As expected, several well-known chemoresistance-related ABC transporters, such as ABCG2 and ABCB1, were significantly upregulated in our established Gem-R cells (Supplementary Fig. S1). Notably, the expression of ABCA8 was also substantially increased at both the mRNA and protein levels in these Gem-R cells (Fig. 1E, F), indicating the involvement of ABCA8 in the acquired chemoresistance of PC. Interestingly, Gem-R cells also exhibited markedly increased migratory and invasive ability (Supplementary Fig. S2a).

### ABCA8 knockdown re-sensitizes Gem-R PC cells to GEM in vitro and in vivo

**Fig. 2:**
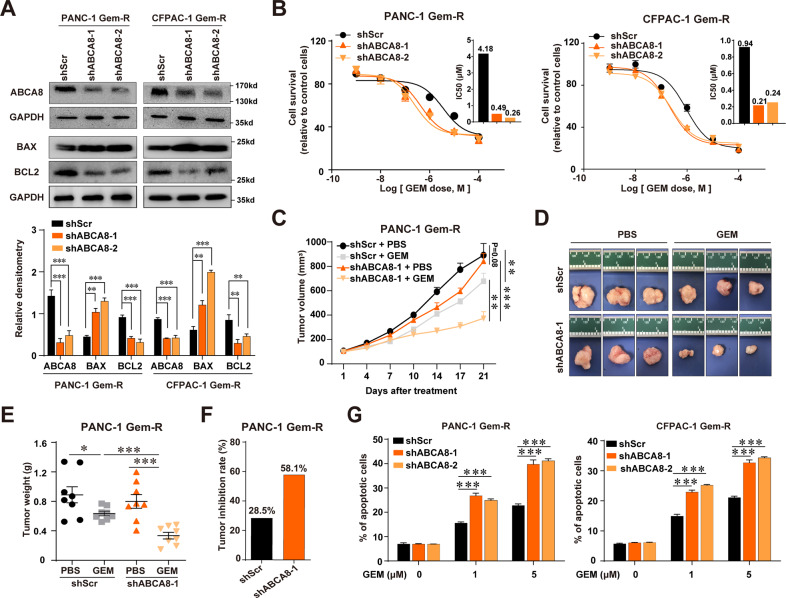
Knockdown of ABCA8 in Gem-R PC cells improves GEM sensitivity in vitro and in vivo. ABCA8 was stably knocked down in PANC-1 or CFPAC-1 Gem-R cells using shRNA targeting the *ABCA8* gene (shABCA8). A scrambled shRNA (shScr) was used as a control. **A** Western blot analyses of ABCA8, BAX, and BCL2 protein expression in shScr or shABCA8 Gem-R cells. The histogram shows the densitometric analysis of the bands (*n* = 3 independent biological repeats). **B** GEM sensitivity of Gem-R shABCA8 cells compared with that of shScr cells was determined by CCK-8 cytotoxicity assays (*n* = 4 independent biological repeats). Histograms show the comparison of IC_50_ values between Gem-R shABCA8 cells and shScr cells. **C**–**F** BALB/c nude mice were subcutaneously injected with PANC-1 Gem-R shScr or shABCA8-1 cells and then treated with GEM (50 mg/kg, 4 times/week) or PBS as a control (*n* = 8 for each group). Growth curves of Gem-R shScr tumors and shABCA8 tumors were shown in **C**. The subcutaneous tumors were collected and weighed at the time of killing (3 weeks after treatment). Three representative tumors for each group are shown in **D**. **E** Comparison of tumor weight upon necropsy between groups. **F** Comparison of the tumor inhibition rate of GEM therapy between PANC-1 Gem-R shScr and shABCA8 tumors. **G** shScr- or shABCA8-transfected Gem-R PANC-1 or CFPAC-1 cells were treated with 1 or 5 μM GEM for 72 h and cell apoptosis was detected by annexin V/7-AAD staining followed by flow cytometry (*n* = 4 independent biological repeats). *P < 0.05, **P < 0.01, and ***P < 0.001.

To determine whether increased ABCA8 expression contributed to acquired chemoresistance, we knocked down ABCA8 in Gem-R cells (Fig. 2A and Supplementary Fig. S3). As shown, the IC_50_ values for GEM were substantially reduced by ABCA8 knockdown in Gem-R cells (Fig. 2B). To further confirm the above findings in vivo, nude mice with established subcutaneous xenograft PANC-1 Gem-R shScr or shABCA8 tumors were treated with GEM. As shown in Fig. 2C–E, GEM treatment resulted in a slight but significant reduction in shScr tumors (tumor weight: 0.89 ± 0.31 g vs. 0.64 ± 0.09 g, *P* < 0.05), whereas the GEM-induced tumor reduction was much more profound in shABCA8 tumors (tumor weight: 0.80 ± 0.26 g vs. 0.33 ± 0.13 g, *P* < 0.001). The tumor inhibition rate of GEM therapy for Gem-R tumors was increased from 28.5% to 58.1% by ABCA8 knockdown (Fig. 2F). These results indicated that ABCA8 contributed to acquired chemoresistance, and that the knockdown of ABCA8 could re-sensitize Gem-R PC cells to GEM therapy.

GEM treatment was able to induce the apoptosis of cancer cells, including PC cells^26^. Here we found that ABCA8 knockdown significantly promoted GEM-induced apoptosis in Gem-R cells (Fig. 2G and Supplementary Fig. S4). BCL2 family members play important roles in anticancer drug-induced cancer cell apoptosis. The pro-survival protein BCL2 confered resistance and the pro-apoptosis protein BAX sensitized PC cells to GEM-induced apoptosis^27^. As expected, knockdown of ABCA8 upregulated BAX and downregulated BCL2 in Gem-R cells (Fig. 2A). In addition, ABCA8 knockdown also decreased the migratory and invasive ability of Gem-R cells (Supplementary Fig. S2b).

### ABCA8 overexpression decreases the sensitivity of PC cells to GEM in vitro and in vivo

**Fig. 3:**
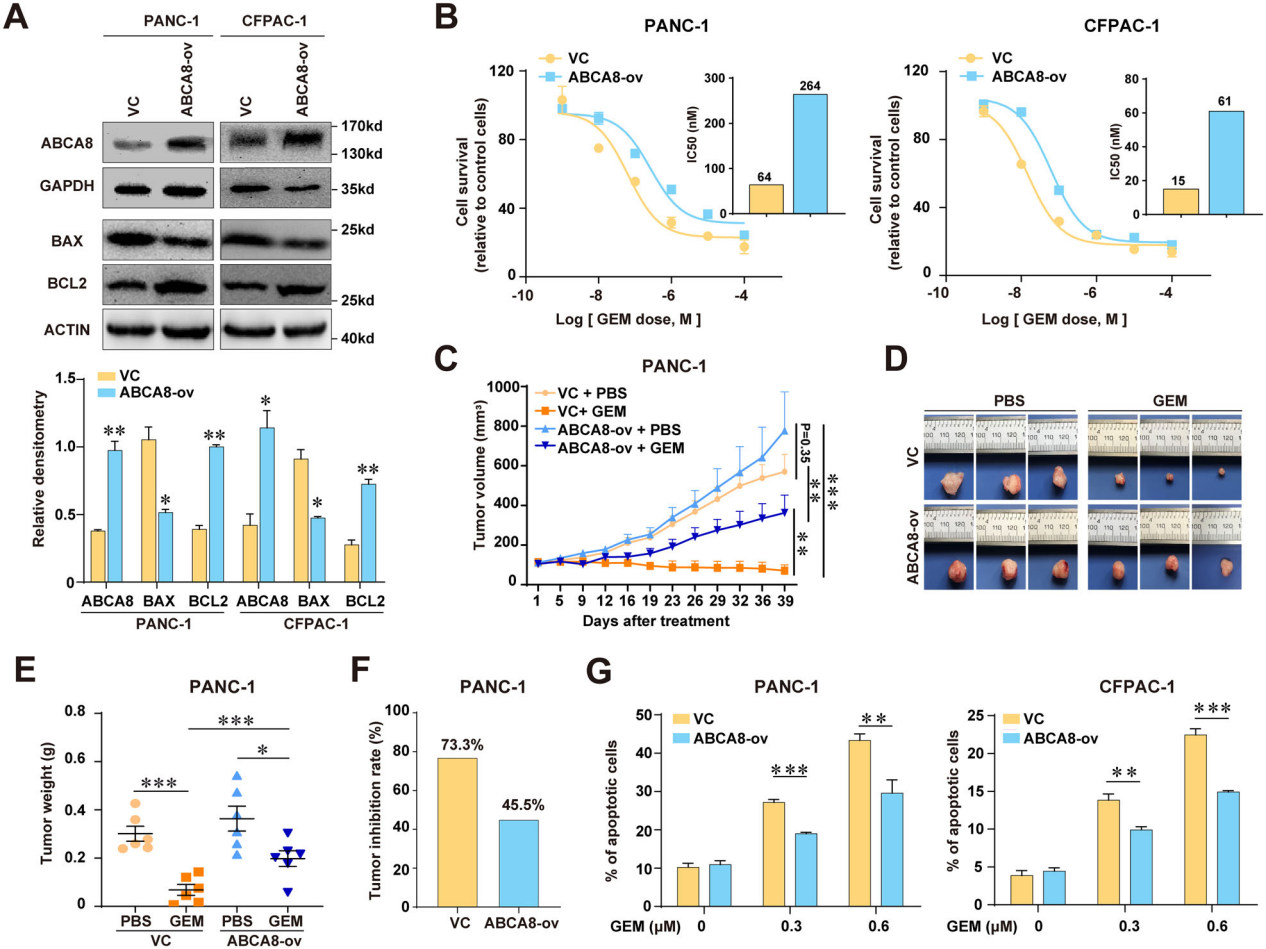
Overexpression of ABCA8 reduces GEM sensitivity in human pancreatic cancer in vitro and in vivo. **A** Expression of ABCA8, BAX, and BCL2 in ABCA8-overexpressing (ABCA8-ov) PANC-1 or CFPAC-1 cells and the corresponding control cells (VC) were examined by western blotting (*n* = 3 independent biological repeats). **B** GEM sensitivity of ABCA8-ov cells compared with control cells was determined by CCK-8 cytotoxicity assays (*n* = 4 independent biological repeats). Histograms show the comparison of IC_50_ values between ABCA8-ov cells and control cell. **C**–**F** BALB/c nude mice were subcutaneously injected with ABCA8-overexpressing PANC-1 cells or control cells and then intraperitoneally treated with GEM (50 mg/kg, twice/week) or PBS (*n* = 6 for each group). Growth curves of ABCA8-overexpressing tumors and control tumors were shown in **C**. The subcutaneous tumors were collected and weighed at the time of killing (5 weeks after treatment). Three representative tumors for each group are shown in **D**. **E** Comparison of tumor weight upon necropsy between groups. **F** Comparison of the tumor inhibition rate for GEM therapy between ABCA8-overexpressing PANC-1 tumors and control tumors. **G** Flow cytometric analysis of GEM-induced apoptosis in ABCA8-ov PANC-1 or CFPAC-1 cells and control cells (*n* = 4 independent biological repeats). *P < 0.05, **P < 0.01, and ***P < 0.001.

To further confirm the functional role of ABCA8 in regulating the chemosensitivity of human PC, we then overexpressed ABCA8 in PANC-1 and CFPAC-1 cells. The overexpression of ABCA8, which was confirmed by western blotting (Fig. 3A), decreased the sensitivity to GEM in vitro. ABCA8-overexpressing PC cells had substantially higher IC_50_ values than control cells (Fig. 3B). ABCA8 overexpression also reduced the 5-FU sensitivity of PC cells (Supplementary Fig. S5). However, it failed to significantly reduce the chemosensitivity of the normal human pancreatic duct epithelial hTERT-HPNE cells (Supplementary Fig. S6). We then evaluated the therapeutic efficacy of GEM treatment on ABCA8-overexpressing tumors in nude mice. As shown in Fig. 3C–E, whereas GEM treatment exhibited a profound inhibitory effect on the growth of control tumors (tumor weight: 0.30 ± 0.07 g vs. 0.07 ± 0.05 g, *P* < 0.001), it failed to achieve comparable therapeutic efficacy for ABCA8-overexpressing tumors (tumor weight: 0.36 ± 0.12 g vs. 0.20 ± 0.07 g, *P* < 0.05). The tumor inhibition rate was decreased from 73.3% in control tumors to 45.5% in ABCA8-overexpressing tumors (Fig. 3F), suggesting that increased ABCA8 expression reduced the sensitivity of human PC cells to GEM therapy.

Meanwhile, ABCA8 overexpression substantially attenuated GEM-induced apoptosis of PC cells (Fig. 3G). Consistently, ABCA8-overexpressing cells exhibited decreased BAX expression and increased BCL2 expression compared with control cells (Fig. 3A). In addition, ABCA8 overexpression significantly enhanced the migration and invasion of PC cells (Supplementary Fig. S2c).

### ABCA8 induces GEM insensitivity and regulates BCL2/BAX balance via ERK signaling in PC cells

**Fig. 4:**
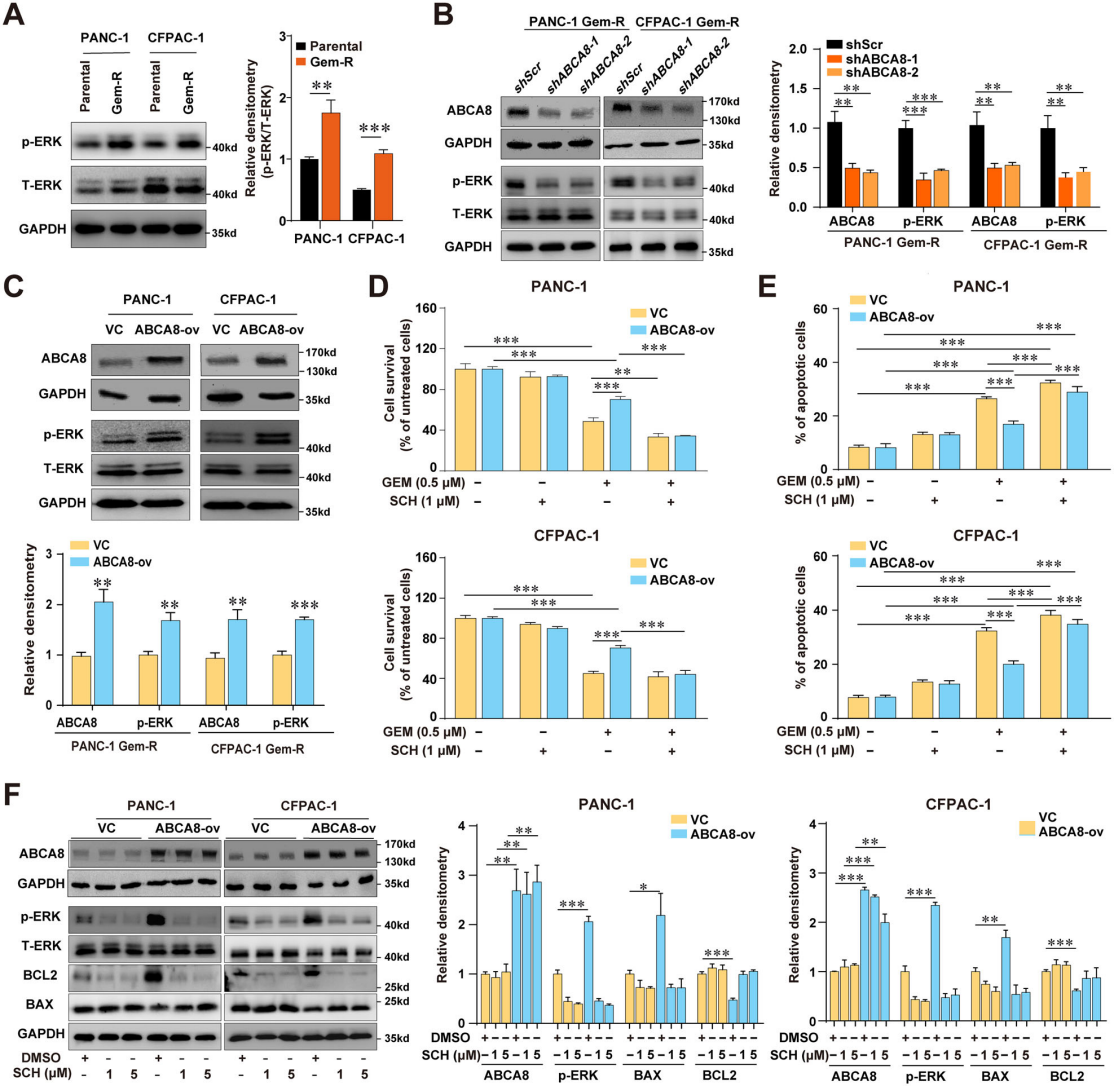
ABCA8 decreases the chemosensitivity of PC cells to GEM via ERK signaling. **A** The expression levels of phosphorylated ERK (p-ERK) and total ERK (T-ERK) proteins in parental and Gem-R pancreatic cancer cells were analyzed by western blotting (*n* = 3 independent biological repeats). **B** Western blot analysis of the effect of ABCA8 knockdown on ERK phosphorylation in Gem-R pancreatic cancer cells (*n* = 3 independent biological repeats). **C** Western blot analysis of the effect of ABCA8 overexpression on ERK phosphorylation in PANC-1 and CFPAC-1 cells (*n* = 3 independent biological repeats). **D** Evaluation of the effect of the ERK inhibitor SCH772984 (SCH) on GEM sensitivity in ABCA8-overexpressing and control (VC) cells by CCK-8 assays after 72 h of treatment. Data are presented relative to the respective untreated controls for ABCA8-overexpressing and control cells (*n* = 4 independent biological repeats). **E** Analysis of the effect of the ERK inhibitor SCH772984 on GEM-induced apoptosis in ABCA8-overexpressing and control cells by flow cytometry (*n* = 4 independent biological repeats). **F** Western blot analysis of the effect of the ERK inhibitor SCH772984 on BAX and BCL2 expression in ABCA8-overexpressing and control cells (*n* = 3 independent biological repeats). ***P < 0.001.

**Fig. 5:**
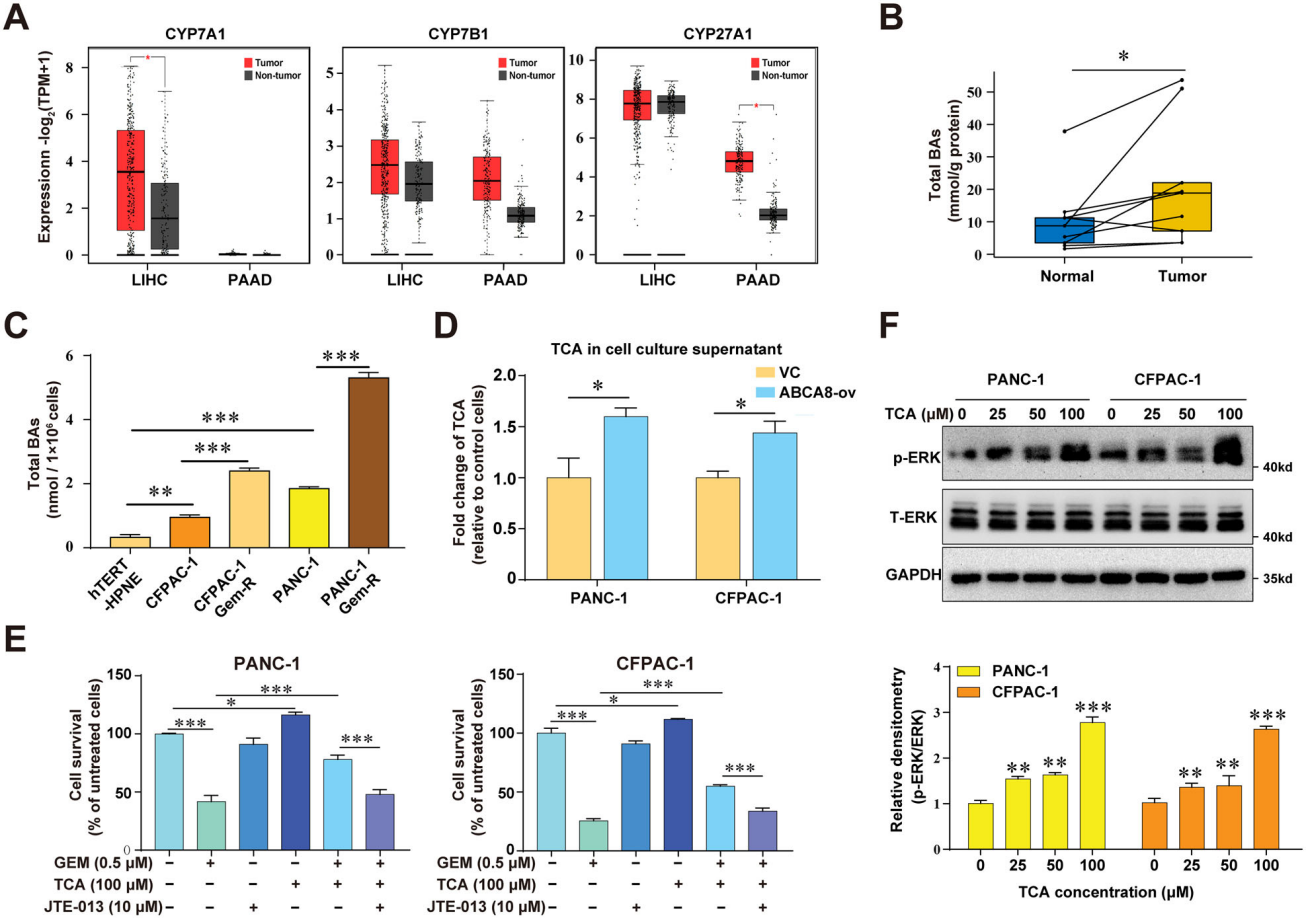
ABCA8 stimulates the efflux of TCA that contributes the GEM resistance in PC cells. **A** The mRNA expression levels of bile acid biosynthesis enzymes CYP7A1, CYP7B1, and CYP27A1 in pancreatic adenocarcinoma (PAAD) (tumor samples: *n* = 179; normal samples: *n* = 171) and liver hepatocellular carcinoma (LIHC) (tumor samples: *n* = 369; normal samples: *n* = 160) were analyzed using the online tool from the GEPIA2 website (http://gepia2.cancer-pku.cn) based on TCGA and GTEx database. **B** Measurement of total BAs in human pancreatic cancer tissues and corresponding adjacent normal tissues (*n* = 9). **C** Measurement of the intracellular total BAs of parental and Gem-R PANC-1 and CFPAC-1 cells, and hTERT-HPNE cells (*n* = 4 independent biological repeats). **D** Measurement of extracellular TCA in the cell culture medium of ABCA8-overexpressing PANC-1 and CFPAC-1 cells and control cells (VC) by a human TCA ELISA kit (*n* = 4 independent biological repeats). **E** Evaluation of the effect of exogenous TCA and/or S1PR2 inhibitor JTE-013 on GEM sensitivity in ABCA8-overexpressing and control (VC) cells by CCK-8 assays after 72 h of treatment (*n* = 4 independent biological repeats). **E** Western blot analysis of ERK phosphorylation in PANC-1 and CFPAC-1 cells treated with the indicated concentrations of exogenous TCA (*n* = 3 independent biological repeats). *P < 0.05, **P < 0.01, and ***P < 0.001.

The activation of extracellular signal-regulated kinase (ERK) signaling activation has been involved in the drug resistance of PC cells. Unsurprisingly, increased ERK phosphorylation was observed in our Gem-R cells (Fig. 5A). Interestingly, the results from Gene Ontology enrichment analysis of The Cancer Genome Atlas (TCGA) data showed that high ABCA8 expression in PC was significantly associated with “positive regulation of ERK1 and ERK2 cascade” (Supplementary Fig. S7), indicating that ERK signaling also contributed to the ABCA8-mediated chemoresistance. This was supported by our observation that ABCA8 knockdown substantially decreased the phosphorylation levels of ERK in Gem-R PC cells (Fig. 4B). In constrast, ABCA8 overexpression increased the ERK phosphorylation (Fig. 4C). Blockade of ERK signaling by its specific ERK inhibitor SCH772984 abrogated ABCA8-induced chemotherapy insensitivity, as evidenced by the comparable GEM-induced cytotoxicity in ABCA8-overexpressing cells to that in control cells in the presence of the ERK-specific inhibitor SCH772984 (Fig. 4D). Furthermore, SCH772984 also reversed the inhibitory effect of ABCA8 overexpression on GEM-induced apoptosis of PANC-1 or CFPAC-1 cells (Fig. 4E) and abolished the expression changes of BAX and BCL2 induced by ABCA8 overexpression (Fig. 4F). These results suggested that ABCA8 mediated the GEM insensitivity of PC cells by activating the ERK pathway.

### ABCA8 stimulates the efflux of TCA that contributes the GEM resistance in PC cells

ABCA8 has been shown to act as a sinusoidal efflux transporter for TCA^19^. TCA is a conjugated primary bile acid (BA). Although BAs are mainly biosynthesized in the liver, they can also be detected in extrahepatic tissues^28^. Interestingly, the analyses of TCGA and GTEx data showed that PC tissues expressed comparable mRNA levels of CYP27A1 and CYP7B1, two rate-limiting enzymes in the alternative (acidic) pathway of BA biosynthesis, as liver tissues (Fig. 5A). To further determine whether BAs also exist in pancreatic tissue, we measured the total BAs in nine paired human PC tissues and adjacent normal tissues. As shown in Fig. 5B, there were detectable levels of total BAs in human pancreatic tissues and the BA concentration in tumor tissue was significantly higher than that in adjacent normal tissues. In addition, there were significantly higher levels of intracellular BAs in PC cells than in normal pancreatic ductal cells (Fig. 5C). The intracellular BA levels were further increased in Gem-R cells compared with parental cells (Fig. 5C), suggesting a possible role of BAs in regulating the chemosensitivity of PC.

As TCA, one of the predominant conjugate forms of BAs in humans^29^, has been demonstrated as an endogenous substrate of ABCA8^19^ and there were significantly higher levels of extracellular TCA in the media of ABCA8-overexpressing cells (Fig. 5D), we further treated PC cells with TCA to determine its functional effect. Extracellular TCA stimulation significantly decreased the sensitivity of both PANC-1 and CFPAC-1 cells to GEM (Fig. 5E). In addition, TCA treatment also led to increased phosphorylation of ERK (Fig. 5F), suggesting that extracellular TCA is involved in regulating the chemosensitivity of PC cells. Further, TCA treatment also led to significant enhancement of migratory and invasive properties of PC cells (Supplementary Fig. S2d).

### ABCA8-induced extracellular accumulation of TCA decreases GEM sensitivity though S1PR2/ERK pathway

**Fig. 6:**
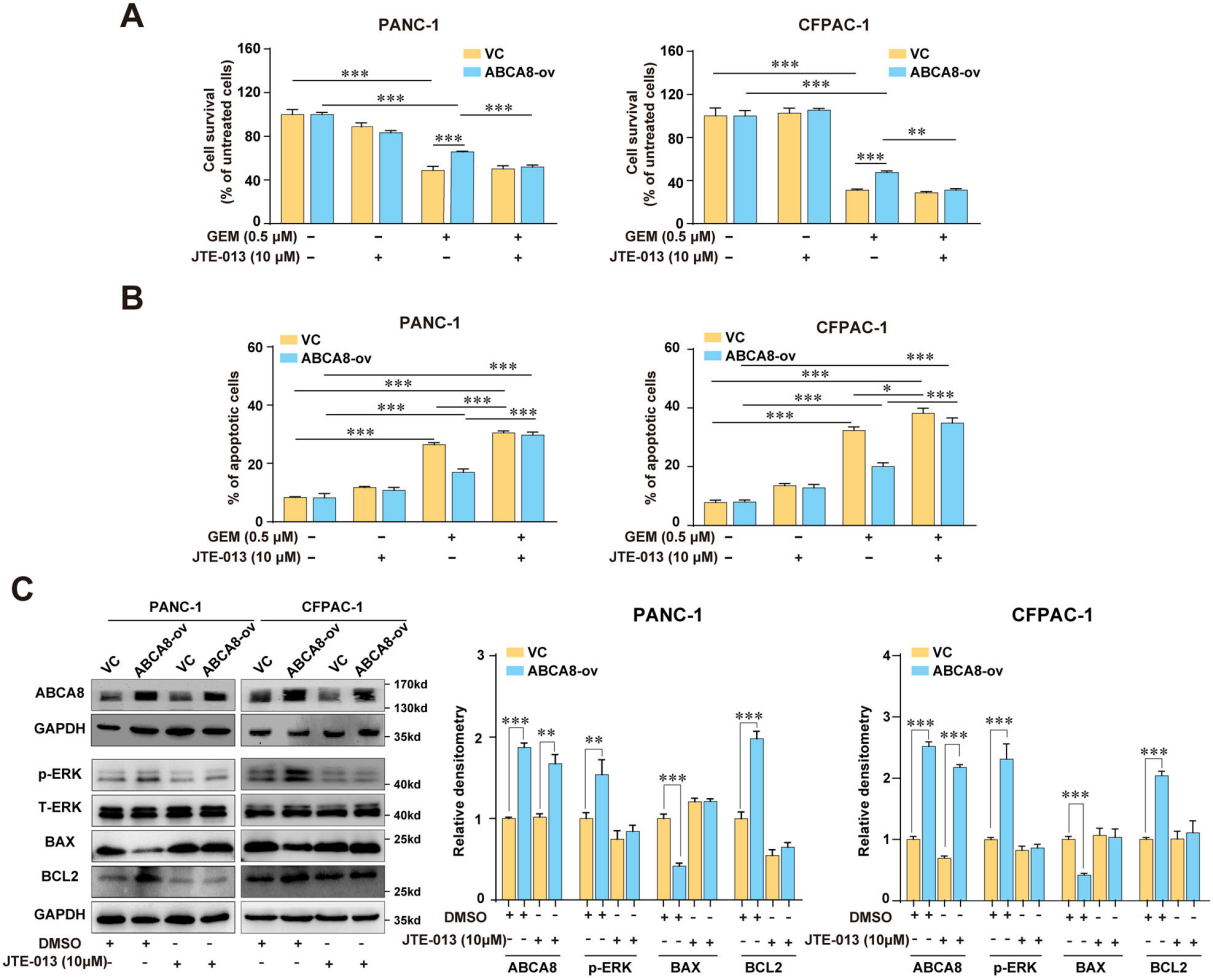
ABCA8-induced extracellular accumulation of TCA contributes to GEM insensitivity by activating SIRP2/ERK signaling. **A**, **B** Analysis of cell survival by CCK-8 assay (**A**) or cell apoptosis levels by flow cytometry (**B**) of ABCA8-overexpressing cells and control cells (VC) in the presence of GEM and/or the S1PR2 inhibitor JTE-013 (*n* = 4 independent biological repeats). Data are presented relative to the respective untreated controls for ABCA8-overexpressing and control cells (**A**). **C** Western blot analysis of the effect of JTE-013 on ERK phosphorylation and the expression of BAX and BCL2 in ABCA8-overexpressing cells and control cells (*n* = 4 independent biological repeats). *P < 0.05, **P < 0.01, and ***P < 0.001.

It has been shown that TCA is a potent signaling agent in the extracellular environment and could act at sphingosine 1-phosphate receptor 2 (S1PR2), a G-protein-coupled receptor, to activate the intracellular signaling pathway^30^. Treatment with the S1PR2-specific inhibitor JTE-013 diminished TCA-induced GEM insensitivity (Fig. 5E), suggesting the involvement of S1PR2 in mediating TCA function in PC cells. Importantly, ABCA8 overexpression failed to decrease the sensitivity to GEM and protect PANC-1 or CFPAC-1 cells against GEM-induced apoptosis in the presense of JTE-013 (Fig. 6A, B). Moreover, blockade of S1PR2 by JTE-013 eliminated the increased phosphorylation of ERK, as well as the expression changes of BAX and BCL2, induced by ABCA8 overexpression (Fig. 6C), suggesting the activation of S1PR2 by TCA as a molecular event upstream of the ERK cascade in ABCA8-overexpressing cells. Together, these results indicated that ABCA8 induces GEM insensitivity of PC cells by pumping TCA out of cells and then activating the S1PR2-ERK axis. Furthermore, inhibition of S1PR2 with JTE-013 also abolished the enhanced migratory and invasive capability induced by ABCA8 overexpression or TCA treatment (Supplementary Fig S2c, d).

## Discussion

**Fig. 7:**
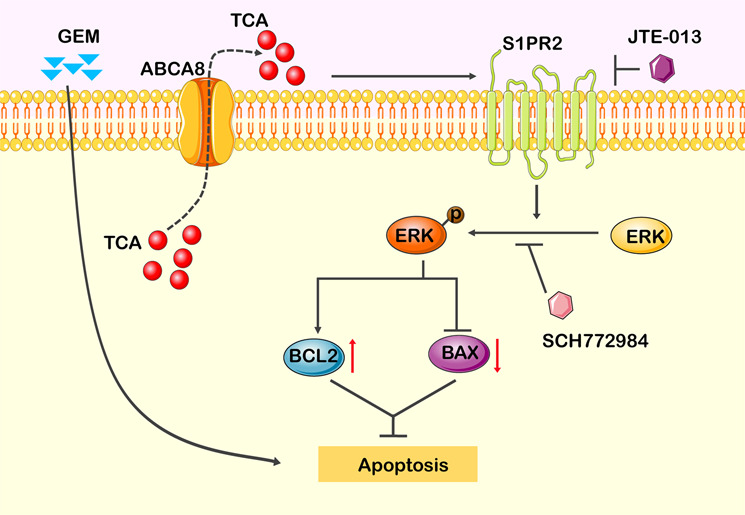
Proposed mechanisms of ABCA8-mediated GEM ineffectiveness in PC. Increased expression of ABCA8 in PC cells fasilitates the efflux of TCA, one conjugated bile acid. Extracellular TCA activates the ERK signaling via the transmembrane receptor S1PR2. The activation of ERK signaling regulates the expressions of the apoptosis-related proteins BAX and BCL2, and then leads to the insensitivity of PC cells to the anticancer drug GEM. Either blockade of S1PR2 by JTE-013 or blockade of ERK signaling by SCH772984 can inhibit the ABCA8-mediated GEM resistance of PC cells.

Recently, the roles of ABCA subfamily transporters in cancers have attracted increasing interest^14^. Regarding ABCA8, the increased expression of this ABCA subfamily transporter has been linked to poor prognosis of cancer patients^24,25^. However, its functional role remains unclear. Here, we highlighted the functional importance of ABCA8 in mediating drug resistance of human PC cells based on a combination of both in vitro and in vivo evidence. Although further clinical research is needed, our results suggest ABCA8 as a potential target for overcoming resistance and improving chemotherapy effectiveness in PC. ABC transporters usually confer chemoresistance due to their ability to efflux anticancer drugs from cancer cells^31,32^. Rather than their role as a drug efflux pump, the current study further presents a novel mechanism of ABC transporter-mediated chemoresistance in PC in which the ABCA subfamily member ABCA8-mediated efflux of TCA contributes to GEM ineffectiveness by activating S1PR2/ERK signaling (Fig. 7).

TCA is a taurine-conjugated form of primary BA. BA is considered to be synthesized mainly in the liver due to the extremely low expression level of the rate-limiting BA biosynthesis enzyme CYP7A1 in extrahepatic tissues^33^. However, BA can also be synthesized via the alternative (acidic) pathway with the rate-limiting enzymes CYP27A1 and CYP7B1, whose expressions are not restricted to the liver^28^. Based on the analyses of TCGA and GTEx data, pancreatic tissues were found to express comparable mRNA levels of CYP27A1 and CYP7B1 as liver tissues despite the extremely low level of CYP7A1. Moreover, we found that there were detectable levels of BAs in pancreatic tissue, as well as in cell lines derived from human normal pancreatic duct or PC. The levels of total BA or CYP27A1 expression were even higher in PC compared to normal pancreatic cells or tissues. Therefore, it is reasonable to believe the existence of BA biosynthesis via the acidic pathway in pancreatic tissue, particularly in PC.

Increasing attention has been paid to the roles of BAs in cancer development and progression. As a predominant BA, TCA was found to promote the invasive growth of cholangiocarcinoma and esophageal adenocarcinoma^34,35^. However, the effect of TCA on the response to anticancer drugs has not yet been studied. Here we showed that extracellular TCA decreased the sensitivity of PC cells to GEM. This observation, together with the fact that ABCA8 facilitates TCA efflux, suggested that ABCA8-mediated extracellular accumulation of TCA contributes to the decreased GEM sensitivity induced by ABCA8. We also noted that, in the normal pancreatic duct epithelial hTERT-HPNE cells that had the markedly lower level of BA biosynthesis than PC cells, ABCA8 overexpression failed to decrease their GEM sensitivity. This observation further strengthened the importance of TCA efflux in ABCA8-induced chemoresistance. It was reported before that S1PR2, which is a member of the sphingosine 1-phosphate G-protein-coupled receptor family^36^, is a transmembrane receptor of extracellular TCA^37,38^. After blockade of S1PR2, we observed a reversal of chemoresistance either in ABCA8-overexpressing cells or in TCA-treated cells. These findings suggested the involvement of TCA and its downstream S1PR2-mediated signaling in the regulation of chemosensitivity and extended our understanding of the role of BAs in cancers.

Our results indicated that TCA-S1PR2-ERK signaling-mediated apoptotic resistance played a role in ABCA8-induced GEM ineffectiveness. Conjugated BAs have been reported to activate the ERK signaling pathway via S1PR2 in rodent hepatocytes^30^ and human cholangiocarcinoma cells^38^. Consistent with those reported, our study also linked TCA-mediated activation of S1PR2 to ERK signaling in PC cells, and demonstrated that SIPR2-ERK is a critical intracellular signal that mediates ABCA8-induced GEM ineffectiveness. The activation of ERK was shown to confer resistance to GEM-induced apoptosis by regulating the expression of the apoptosis-related proteins BCL2 and BAX^27^. In ABCA8-overexpressing PC cells, the activation of ERK was associated not only with apoptosis resistance but also with the expression changes of BCL2 and BAX. Notably, ERK signaling is involved in resistance to other anticancer drugs in addition to GEM^39–41^. In fact, overexpression of ABCA8 in PC cells also reduced their sensitivity to 5-FU, suggesting the involvement of ABCA8-TCA-S1PR2-ERK pathway in the development of multidrug resistance in PC cells.

An increasing body of evidence indicates that chemotherapy can increase the metastatic potential of cancer cells^42^. In particular, GEM treatment has been shown to enhance the cell migration and invasiveness of PC and lung cancer^43,44^. These findings were supported by our observation that Gem-R cells exhibited markedly increased migratory and invasive ability in this study. Interestingly, this study further found that ABCA8 overexpression could enhance the migration and invasion of PC cells and suggested the contribution of TCA-S1PR2 pathway to ABCA8-induced cell migration and invasion. Given that ABCA8 was significantly upregulated by GEM, we can reasonably speculate that ABCA8-TCA-S1PR2 axis might serve as an additional mechanism of GEM therapy-induced metastasis in PC, which awaits further studies to confirm.

In conclusion, our study revealed the role of ABCA8-mediated TCA efflux in regulating PC cells sensitivity to chemotherapy and characterized the TCA-S1PR2-ERK pathway underlying ABCA8-induced GEM ineffectiveness. These findings suggest that targeting ABCA8 or the TCA-S1PR2-ERK pathway represents new strategies for improving the effectiveness of and overcoming the resistance to chemotherapy in PC.

## Materials and methods

### Cell lines and culture

Human PC cell lines (PANC-1, CFPAC-1, BxPC-3, AsPC-1, MIA PaCa-2) and immortalized normal pancreatic duct epithelial hTERT-HPNE cells were obtained from the American Type Culture Collection (Manassas, VA, USA). Cells were cultured in Dulbecco’s modified Eagle’s medium (hTERT-HPNE, PANC-1, MIA PaCa-2), RPMI-1640 (CFPAC-1, BxPC-3), or IMDM (AsPC-1) medium supplemented with 10% fetal bovine serum (Thermo Scientific, Waltham, MA, USA) in a humidified atmosphere of 5% CO_2_ at 37 °C. All cell lines were authenticated using standard short tandem repeat testing by Biowing Applied Biotechnology, Co., Ltd (Shanghai, China). For details about the generation of Gem-R cells, ABCA8-overexpressing cells, or ABCA8-knockdown cells, see Supplementary Materials and Methods.

### Quantitative real-time PCR

RNA isolation from GEM-treated or Gem-R PC cells and quantitative real-time PCR analysis of ABC transporter expression were performed according to our previous description^45^. The relative expression of the target genes was normalized to *GAPDH*. The primer sequences are provided in Supplementary Table S1.

### Western blotting

Western blot analysis was performed according to the procedure described previously^45^. The antibodies used for western blotting are shown in Supplementary Table S2. All western blottings were quantified using ImageJ software and the quantitative results were presented as the relative expression levels of target proteins normalized to the corresponding loading controls or pan-protein levels. The western blotting images shown are representative of three independent experiments.

### Cell viability and cell apoptosis assays

Cell viability was monitored using a CCK-8 (Dojindo, Kumamoto, Japan) and cell apoptosis was analyzed using the PE Annexin V Apoptosis Detection Kit (BioLegend, San Diego, CA, USA) according to the corresponding manufacturer’s protocols. For details about the procedure of these assays, see Supplementary Materials and Methods.

### Animal experiments

Male athymic BALB/c nude mice were used in this study and maintained under specific pathogen-free conditions. Four-week-old nude mice were subcutaneously inoculated with cancer cells (5 × 10^6^ cells/mouse) and the tumor size was monitored. When the tumor volume reached 100 mm^3^, the nude mice were randomly assigned to groups and treated with vehicle or GEM (50 mg/kg, 4 times/week for ABCA8 knockdown experiments and 50 mg/kg, 2 times/week for ABCA8 overexpression experiments). Four or 8 weeks after tumor inoculation, mice were killed and the tumors were excised and weighed. The tumor inhibition rate was calculated using the following formula: inhibition rate (%) = (mean tumor weight of phosphate-buffered saline (PBS)-treated group − mean tumor weight of the GEM-treated group)/mean tumor weight of PBS-treated group. All animal experiments were conducted double-blind and performed according to the protocols approved by the Medical Experimental Animal Care Commission at the Shanghai Cancer Institute.

### BA and TCA measurements

For BA measurement in PC tissues, nine paired PC and adjacent normal tissues were collected from patients pathologically diagnosed with PC in the Third Hospital Affiliated to Nantong University from October 2019 to January 2020. Informed consent was obtained from each patient prior to sample collection. This study was approved by the Ethics Committee of the Third Hospital Affiliated to Nantong University.

The concentration of total BAs was determined by the Total Bile Acids Assay Kit (BioVision, Mountain View, CA, USA) and the concentration of TCA was determined by using the Human Taurocholic Acid Detection ELISA kit (Enzyme-linked Biotechnology, Shanghai, China) according to the corresponding manufacturer’s guidelines. For details about the procedure of these assays, see Supplementary Materials and Methods.

### Statistical analysis

Quantitative data are represented as the mean ± SEM of at least three independent repeated experiments. GraphPad Prism 7.0 software was used to perform statistical analysis. The significance of differences was assessed by Student’s *t*-test for single comparisons or by analysis of variance with the Tukey’s test for multiple comparisons. *P*-values < 0.05 were considered statistically significant.

## Supplementary information

Supplementary Materials and Methods

Supplementary Table S1

Supplementary Table S2

Supplementary Table S3

Supplementary Figure Legends

Supplementary Figure S1

Supplementary Figure S2

Suppelmentary Figure S3

Supplementary Figure S4

Supplementary Figure S5

Supplementary Figure S6

Supplementary Figure S7
