# Supplementary material for: ABCA8-mediated efflux of taurocholic acid contributes to gemcitabine insensitivity in human pancreatic cancer via the S1PR2-ERK pathway
Source: Cell Death Discov. 2021 Jan 11;7:6. doi: 10.1038/s41420-020-00390-z (PMC7801517; doi:10.1038/s41420-020-00390-z)
Supplement: Supplementary file 1 — Supplementary Materials and Methods [file 41420_2020_390_MOESM1_ESM.docx]

**Supplementary Materials and Methods**

***Generation of Gem-R cells***

To create Gem-R human PC cells, PANC-1 or CFPAC-1 cells were first cultured in medium containing 10 nM GEM, and then the concentration of GEM was increased stepwise. The whole process continued for 4 months, and the Gem-R cells showed an over 50-fold increase in resistance to GEM compared with parental cells.

***Generation of ABCA8-overexpressing or ABCA8-knockdown cells***

To overexpress ABCA8 in PC cells, PANC-1 cells or CFPAC-1 cells were infected with recombinant lentivirus carrying the human ABCA8 gene at a multiplicity of infection (MOI) of 10. PANC-1 cells or CFPAC-1 cells were infected with an empty virus and used as control cells for ABCA8 overexpression experiments. To knockdown ABCA8 in Gem-R cells, PANC-1 Gem-R or CFPAC-1 Gem-R cells were infected with lentivirus carrying an shRNA sequence targeting ABCA8 at an MOI of 10. The corresponding Gem-R cells infected with lentivirus containing a scrambled shRNA were used as control cells for ABCA8 knockdown experiments. Sequences of shRNAs are provided in Supplementary Table S3. All recombinant lentiviruses were purchased from Shanghai GeneChem Co. (Shanghai, China).

***Cell apoptosis assay***

Cells were pretreated with or without the ERK inhibitor SCH772984 (Cell Signaling Technology, Danvers, MA, USA) or the S1PR2 inhibitor JTE-013 (Selleck Chemicals, Houston, TX, USA) for 2 hr, followed by GEM treatment for 72 hr. Then, the apoptosis of treated cells was analyzed using the PE Annexin V Apoptosis Detection Kit (BioLegend, San Diego, CA, USA). Annexin V-positive cells were considered apoptotic cells.

***Cell viability analysis***

A total of 8,000 cells/well were plated in 96-well plates 12 hr before the treatment. The cells were then treated with the indicated agents for 72 hr. At the end of the treatment, cell viability was monitored using a CCK8 (Dojindo, Kumamoto, Japan) according to the manufacturer’s protocol. Half-maximal inhibitory concentration (IC_50_) values were calculated by non-linear regression using GraphPad Prism 7.0 (GraphPad Software Inc., San Diego, CA, USA).

***Total BA measurement***

Twenty-fifty micrograms of tumor tissues or adjacent normal tissues were homogenized in PBS on ice. In parallel, human pancreatic cancer cells or hTERT-HPNE cells (3×10^6^) were suspended in 200 μL PBS, and lysed by the freeze-thaw methods. After centrifugation at 10,000 g for 5 min, the supernatant was subjected to BA measurement. The concentration of total BAs was determined by the Total Bile Acids Assay Kit (BioVision, Mountain View, CA, USA) according to the manufacturer’s guidelines.

***TCA measurement***

ABCA8–overexpressing cells or control cells were seeded in a 6-cm dish (3×10^6^ cells/dish). After overnight culture in complete media, the media was changed to FBS-free and phenol red-free media (1.5 ml/dish), and the cells were cultured for an additional 24 hr. Then, the cell culture supernatant was carefully collected, and the concentration of TCA in the supernatant was determined by using the Human Taurocholic Acid Detection ELISA kit (Enzyme-linked Biotechnology, Shanghai, China) according to the manufacturer’s guidelines.

***Cell migration and invasion assays***

For the *in vitro* tumor cell migration and invasion studies, cells were suspended in serum-free medium and plated in the top chamber of the non-coated culture inserts (BD Biosciences, Bedford, MA, USA) for the migration assay (5 × 10^4^ cells/insert) or the Matrigel-coated inserts for the invasion assay (1 × 10^5^ cells/insert). The lower chamber contained medium with 10% FBS as a chemoattractant. After 16 hr of incubation, the cells on the lower surface of the membrane were stained with crystal violet. The cell migration or invasion ability was quantitated by counting the number of crystal violet-stained cells. In some of the experiments, the cells were pretreated with TCA or/and the S1PR2 inhibitor JTE-013 for 16 hr, and then subjected to transwell migration or invasion assay. During the migration/invasion assays, TCA or JTE-013was added to the medium in both top and bottom chambers.
