# Supplementary material for: ABCA8-mediated efflux of taurocholic acid contributes to gemcitabine insensitivity in human pancreatic cancer via the S1PR2-ERK pathway
Source: Cell Death Discov. 2021 Jan 11;7:6. doi: 10.1038/s41420-020-00390-z (PMC7801517; doi:10.1038/s41420-020-00390-z)
Supplement: Supplementary file 2 — Supplementary Table S1 [file 41420_2020_390_MOESM2_ESM.docx]

**Supplementary Table S1 Primers used in quantitative real-time PCR analysis.**

| **Gene** | **Direction** | **Primer sequence (5’-3’)** |
| --- | --- | --- |
| *ABCA8* | Forward | 5'- GGCCCTTTTCTTGGCACTTG-3' |
|  | Reverse | 5'- CAGGCCGGTGAGGAAAGATT-3' |
| *ABCB1* | Forward | 5'- CCCATCATTGCAATAGCAGG-3' |
|  | Reverse | 5'- GTTCAAACTTCTGCTCCTGA-3' |
| *ABCG2* | Forward | 5'- TGGCTGTCATGGCTTCAGTACT-3' |
|  | Reverse | 5'- CATTATGCTGCAAAGCCGTAAA-3' |
| *GAPDH* | Forward | 5'- TGACGTGGACATCCGCAAAG-3' |
|  | Reverse | 5'- CTGGAAGGTGGACAGCGAGG-3' |
