# Supplementary material for: ABCA8-mediated efflux of taurocholic acid contributes to gemcitabine insensitivity in human pancreatic cancer via the S1PR2-ERK pathway
Source: Cell Death Discov. 2021 Jan 11;7:6. doi: 10.1038/s41420-020-00390-z (PMC7801517; doi:10.1038/s41420-020-00390-z)
Supplement: Supplementary file 3 — Supplementary Table S2 [file 41420_2020_390_MOESM3_ESM.docx]

**Supplementary Table S2 Antibodies used in this study.**

| **Antibody** | **Dilution** | **Product Number** | **Company** |
| --- | --- | --- | --- |
| ABCA8 | 1:1000 | #[45290002](https://www.novusbio.com/products/abca8-antibody_45290002) | Novus |
| p-ERK | 1:1000 | #4695 | Cell Signaling Technology |
| T-ERK | 1:1000 | #4370 | Cell Signaling Technology |
| BCL2 | 1:2000 | #12789-1-AP | ProteinTech |
| BAX | 1:2000 | #50599-2-lg | ProteinTech |
| GAPDH | 1:50000 | # G8795 | Sigma |
| ACTIN | 1:50000 | # A1978 | Sigma |
| Goat anti-Mouse IgG | 1:500000 | #12-349 | Sigma |
| Goat anti-Rabbit IgG | 1:500000 | #12-348 | Sigma |
