# Supplementary material for: ABCA8-mediated efflux of taurocholic acid contributes to gemcitabine insensitivity in human pancreatic cancer via the S1PR2-ERK pathway
Source: Cell Death Discov. 2021 Jan 11;7:6. doi: 10.1038/s41420-020-00390-z (PMC7801517; doi:10.1038/s41420-020-00390-z)
Supplement: Supplementary file 4 — Supplementary Table S3 [file 41420_2020_390_MOESM4_ESM.docx]

**Supplementary Table S3 shRNAs used in this study.**

| **Name** | **Sequence (5’-3’)** |
| --- | --- |
| shScr | 5'- TTCTCCGAACGTGTCACGT-3' |
| shABCA8-1 | 5'- CGAAGGCCAAATCACTGCAAT-3' |
| shABCA8-2 | 5'- CATGGGTCATAGTATCTGATA-3' |
| shABCA8-3 | 5'- ccAGTTCTTATGGACATTGTT-3' |
