# Supplementary material for: ABCA8-mediated efflux of taurocholic acid contributes to gemcitabine insensitivity in human pancreatic cancer via the S1PR2-ERK pathway
Source: Cell Death Discov. 2021 Jan 11;7:6. doi: 10.1038/s41420-020-00390-z (PMC7801517; doi:10.1038/s41420-020-00390-z)
Supplement: Supplementary file 5 — Supplementary Figure Legends [file 41420_2020_390_MOESM5_ESM.docx]

**Supplementary Figure Legends**

**Supplementary Fig. S1 ABCG2 and ABCB1 are upregulated in Gem-R PC cells.**

The mRNA expression of ABCG2 and ABCB1 in Gem-R PANC-1 cells and CFPAC-1 cells was measured by quantitative real-time PCR. **P* < 0.05, ** *P* < 0.01.­­­

**Supplementary Fig. S2 The effect of ABCA8 and TCA on the *in vitro* migration and invasion of PC cells.**

**a** The *in vitro* cell migration or invasion of Gem-R PANC-1 or CFPAC-1 cells were determined by transwell assays. Representative images are shown in the left panel (200×). The right panel shows the comparison of the migration and invasion abilities between parental and Gem-R cells. **b-d** Transwell assays were also used to determine the effect of ABCA8 knockdown (**b**), ABCA8 overexpression (**c**), TCA treatment (**d**) on the migration and invasion of parental or Gem-R PANC-1 or CFPAC-1 cells in the absent or present of the S1PR2 inhibitor JTE-013. **P* < 0.05, ** *P* < 0.01, and *** *P* < 0.001.

**Supplementary Fig. S3 Quantitative real-time PCR analysis of the ABCA8 mRNA expression in ABCA8 knockdown cells.**

ABCA8 was stably knocked down in PANC-1 or CFPAC-1 Gem-R cells using shRNA targeting the ABCA8 gene (shABCA8). A scrambled shRNA (shScr) was used as a control. The knockdown of ABCA8 mRNA expression was confirmed by quantitative real-time PCR. Note that shABCA8-1 and shABCA8-2 were used for subsequent experiments due to that shABCA8-3 failed to significantly decrease the mRNA expression of ABCA8 in both cells. **P* < 0.05, *** *P* < 0.001.

**Supplementary Fig. S4 Representative panels of flow cytometric analysis of the apoptosis of Gem-R cells.**

Scrambled shRNA (shScr)- or ABCA8-targeted shRNA (shABCA8)-transfected Gem-R CFPAC-1 cells were treated with 1 or 5 μM GEM and cell apoptosis was detected by annexin V/7-AAD staining followed by flow cytometry. Representative panels of flow cytometric analysis of the apoptosis of Gem-R cells are shown here.

**Supplementary Fig. S5 Overexpression of ABCA8 in PC cells reduces 5-fluorouracil sensitivity *in vitro*.**

ABCA8-overexpressing (ABCA8-ov) PANC-1 or CFPAC-1 cells and corresponding control cells (VC) were treated with 5-fluorouracil (5-FU) at the indicated concentrations and cell survival was determined by CCK-8 assays after 5-FU treatment for 72 hr. *** *P* < 0.001.

**Supplementary Fig. S6 Overexpression of ABCA8 cannot reduce GEM sensitivity in nonmalignant human pancreatic duct epithelial** **hTERT-HPNE cells.**

**a** Expression of ABCA8 in ABCA8-overexpressing (ABCA8-ov) hTERT-HPNE cells and the corresponding control cells (VC) was examined by western blotting (n = 3 independent biological repeats). **b** GEM sensitivity of ABCA8-ov cells hTERT-HPNE compared with control cells was determined by CCK-8 cytotoxicity assays (n = 3 independent biological repeats). Histograms show the comparison of IC_50_ values between ABCA8-ov cells and control cells.

**Supplementary Fig. S7 Bioinformatics analysis of possible functions of ABCA8 using mRNA data of pancreatic adenocarcinoma from TCGA database.**

**a** Screening process of differentially expressed genes between pancreatic adenocarcinoma samples with high ABCA8 levels (ABCA8-High, n=10) and that with low ABCA8 levels (ABCA8-Low, n=10) from TCGA database. **b** Comparison of ABCA8 expression between ABCA8-High samples and ABCA8-Low samples. **c** Differentially expressed genes between ABCA8-High samples and ABCA8-Low samples were selected by volcano plot filtering using R software (fold change > 3 and P < 0.01). A total of 810 genes were significantly upregulated, and 190 genes were significantly downregulated. **d** Major pathways identified from GO enrichment analysis of upregulated genes using the web-accessible functional annotation tool from the Database for Annotation, Visualization, and Integrated Discovery (DAVID) (<http://david.abcc.ncifcrf.gov>).
