# Supplementary figures and images for: ABCA8-mediated efflux of taurocholic acid contributes to gemcitabine insensitivity in human pancreatic cancer via the S1PR2-ERK pathway
Source: Cell Death Discov. 2021 Jan 11;7:6. doi: 10.1038/s41420-020-00390-z (PMC7801517; doi:10.1038/s41420-020-00390-z)

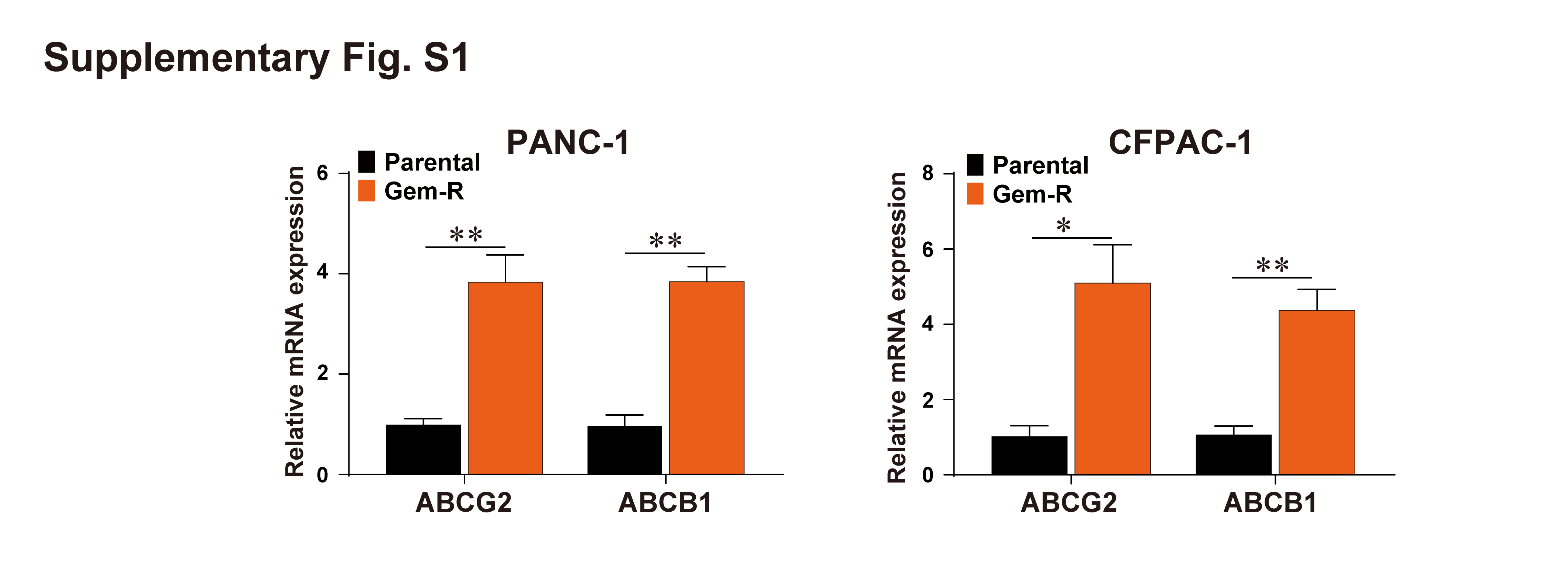

Supplement: Supplementary file 6 — Supplementary Figure S1 [file 41420_2020_390_MOESM6_ESM.tif]

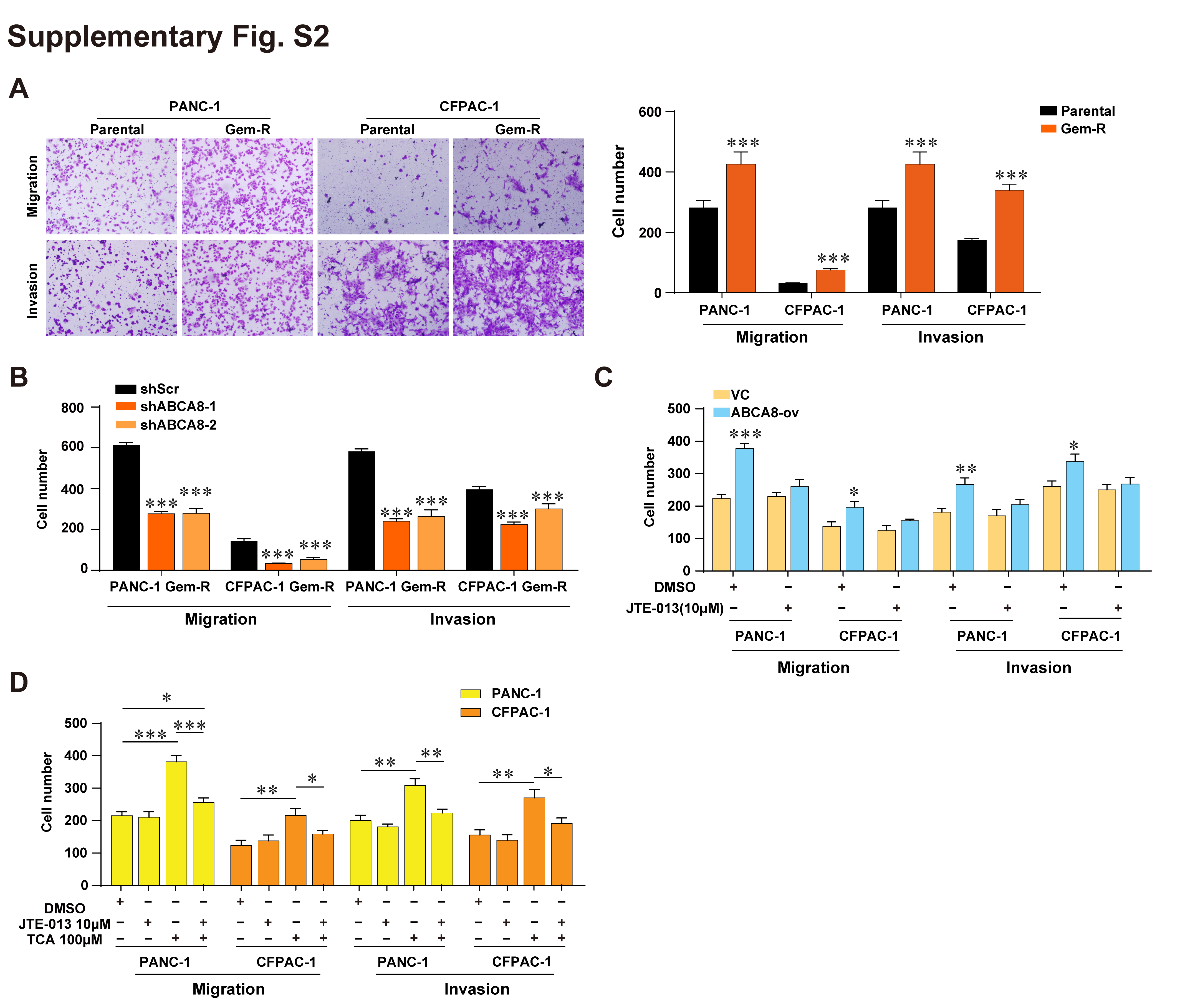

Supplement: Supplementary file 7 — Supplementary Figure S2 [file 41420_2020_390_MOESM7_ESM.tif]

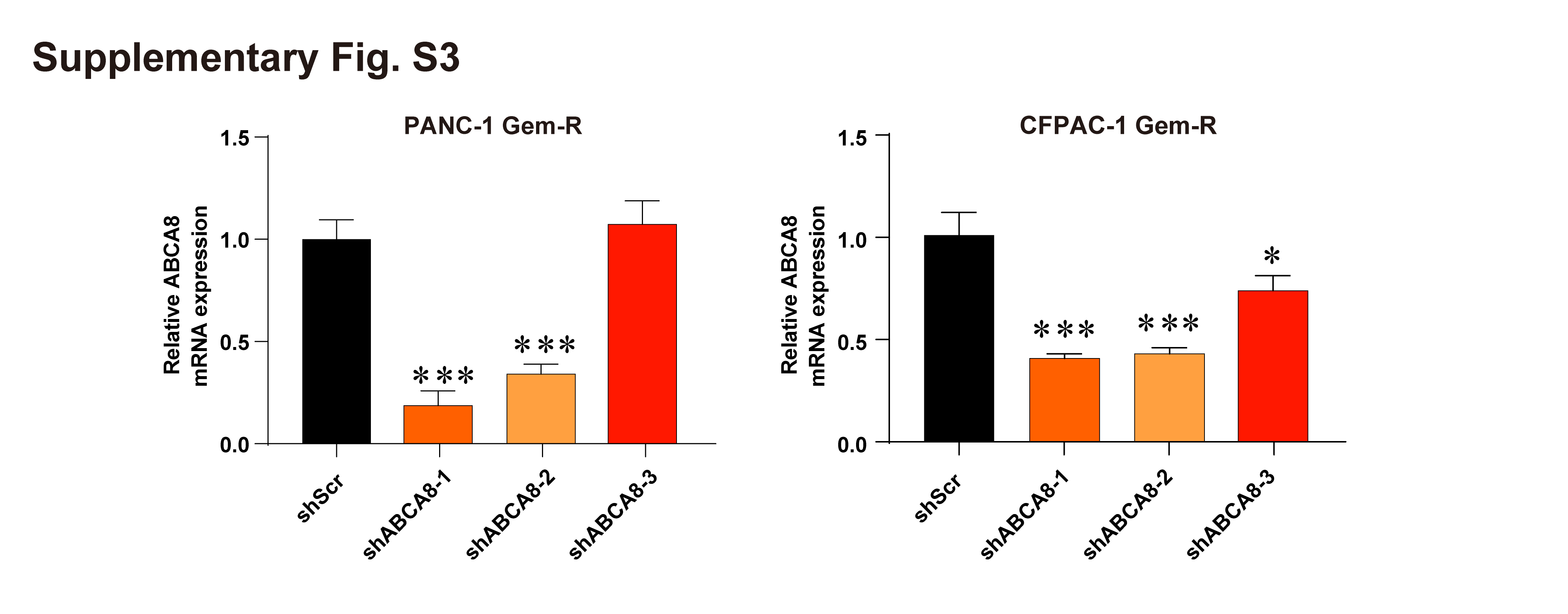

Supplement: Supplementary file 8 — Suppelmentary Figure S3 [file 41420_2020_390_MOESM8_ESM.tif]

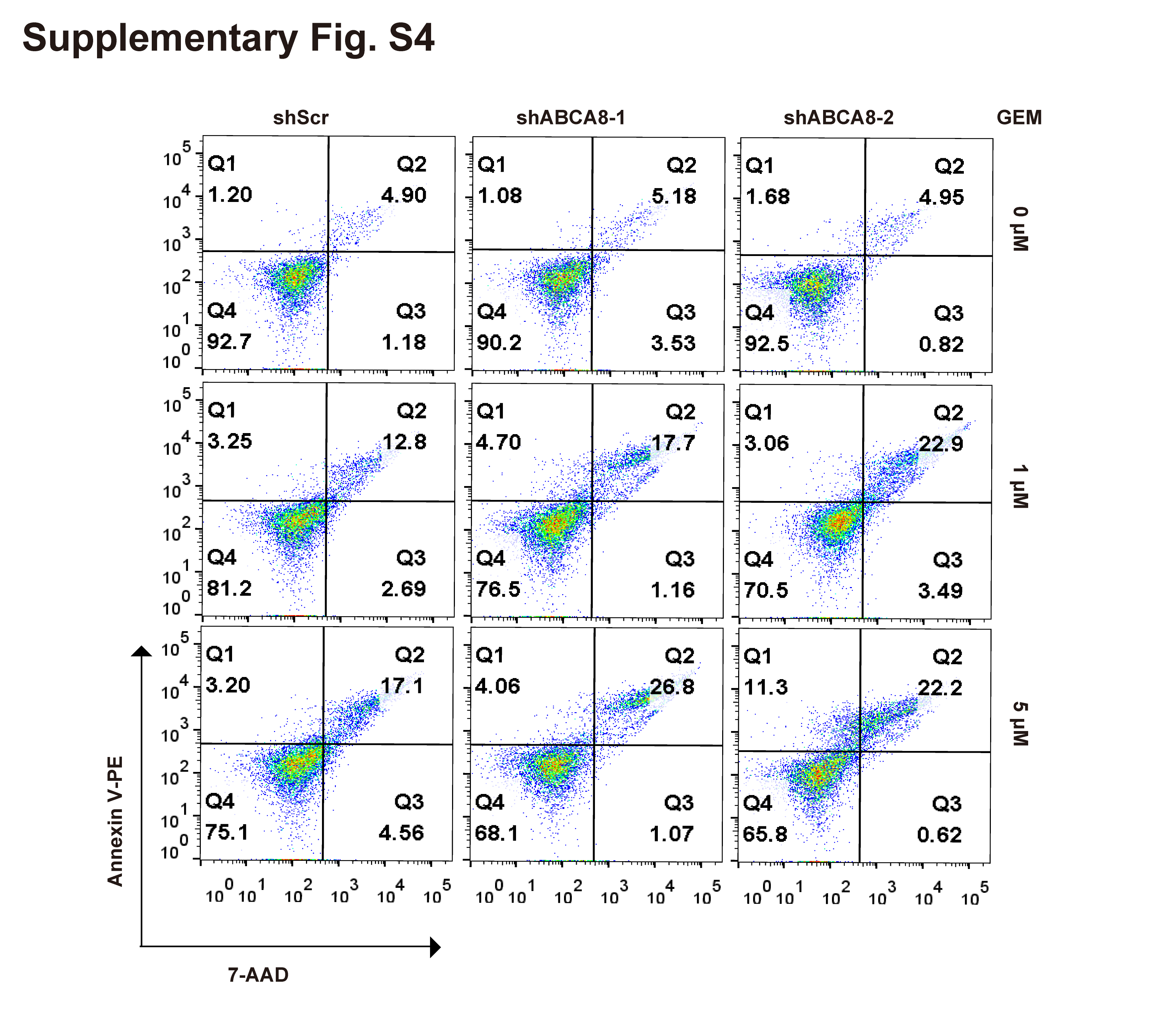

Supplement: Supplementary file 9 — Supplementary Figure S4 [file 41420_2020_390_MOESM9_ESM.tif]

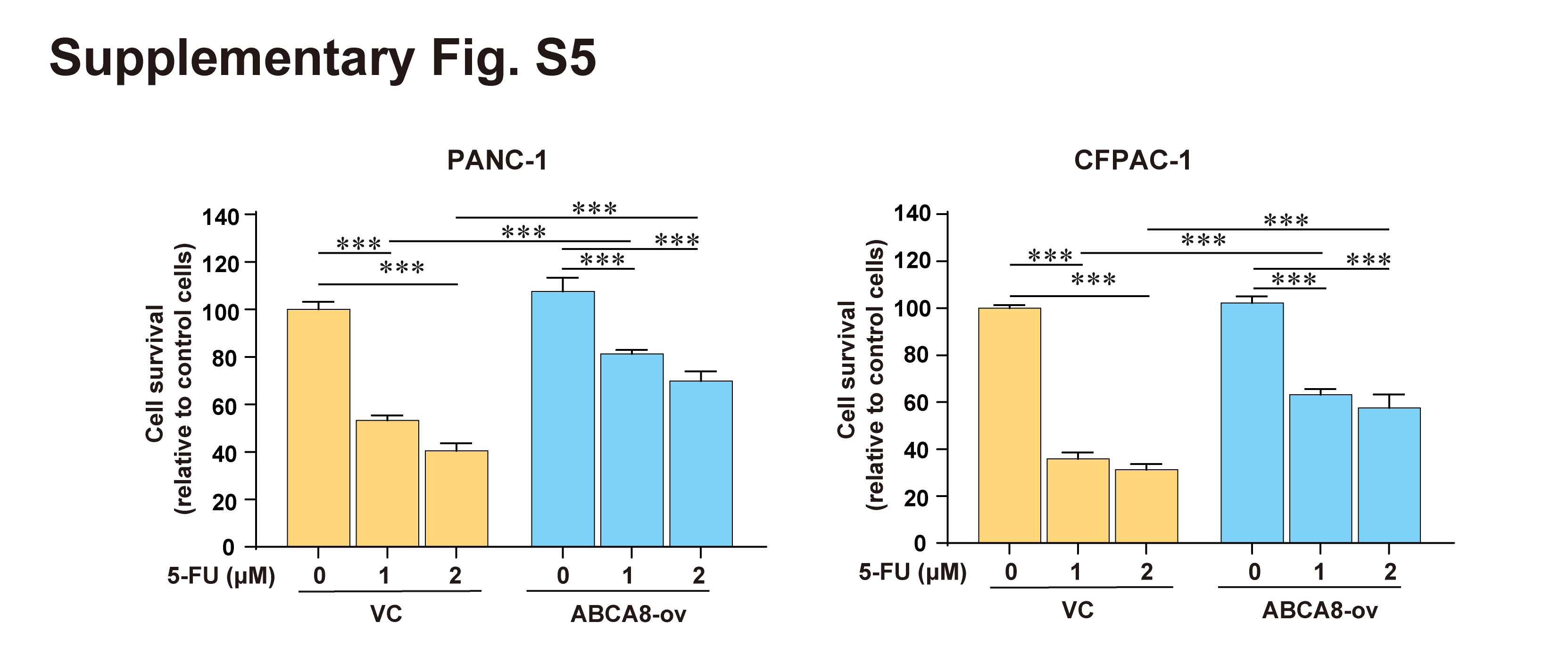

Supplement: Supplementary file 10 — Supplementary Figure S5 [file 41420_2020_390_MOESM10_ESM.tif]

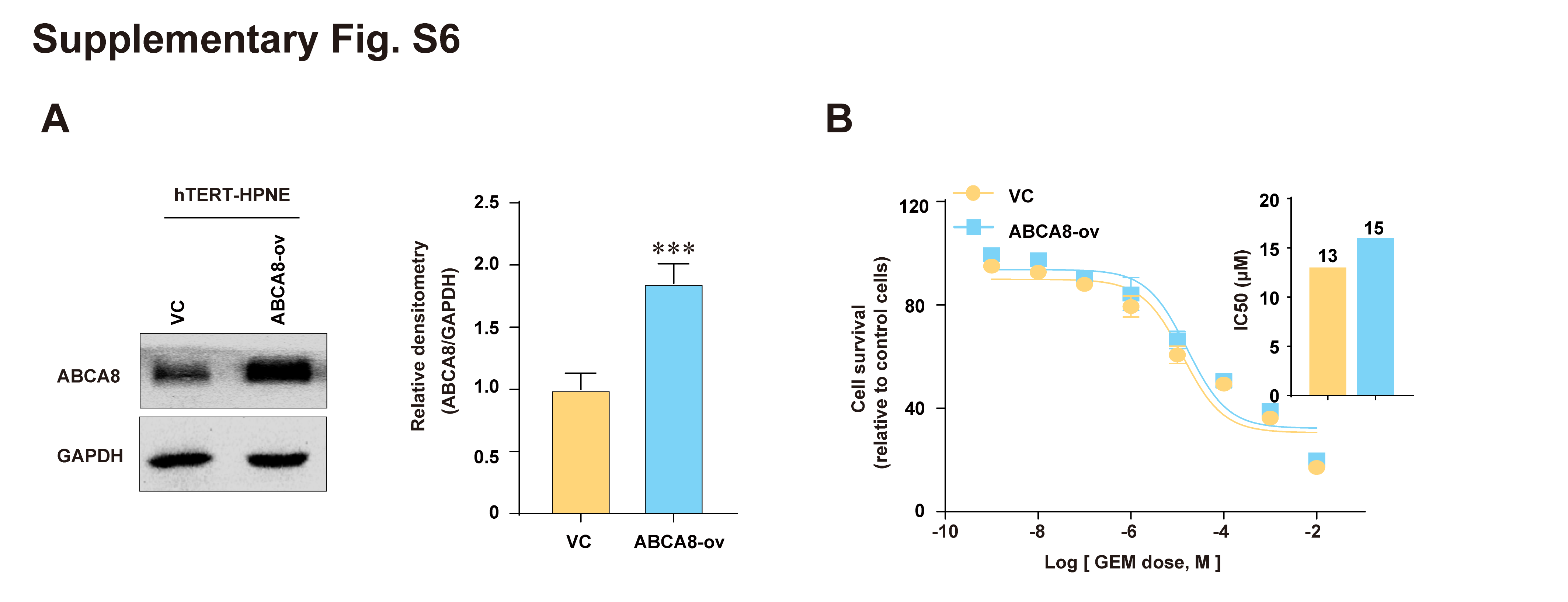

Supplement: Supplementary file 11 — Supplementary Figure S6 [file 41420_2020_390_MOESM11_ESM.tif]

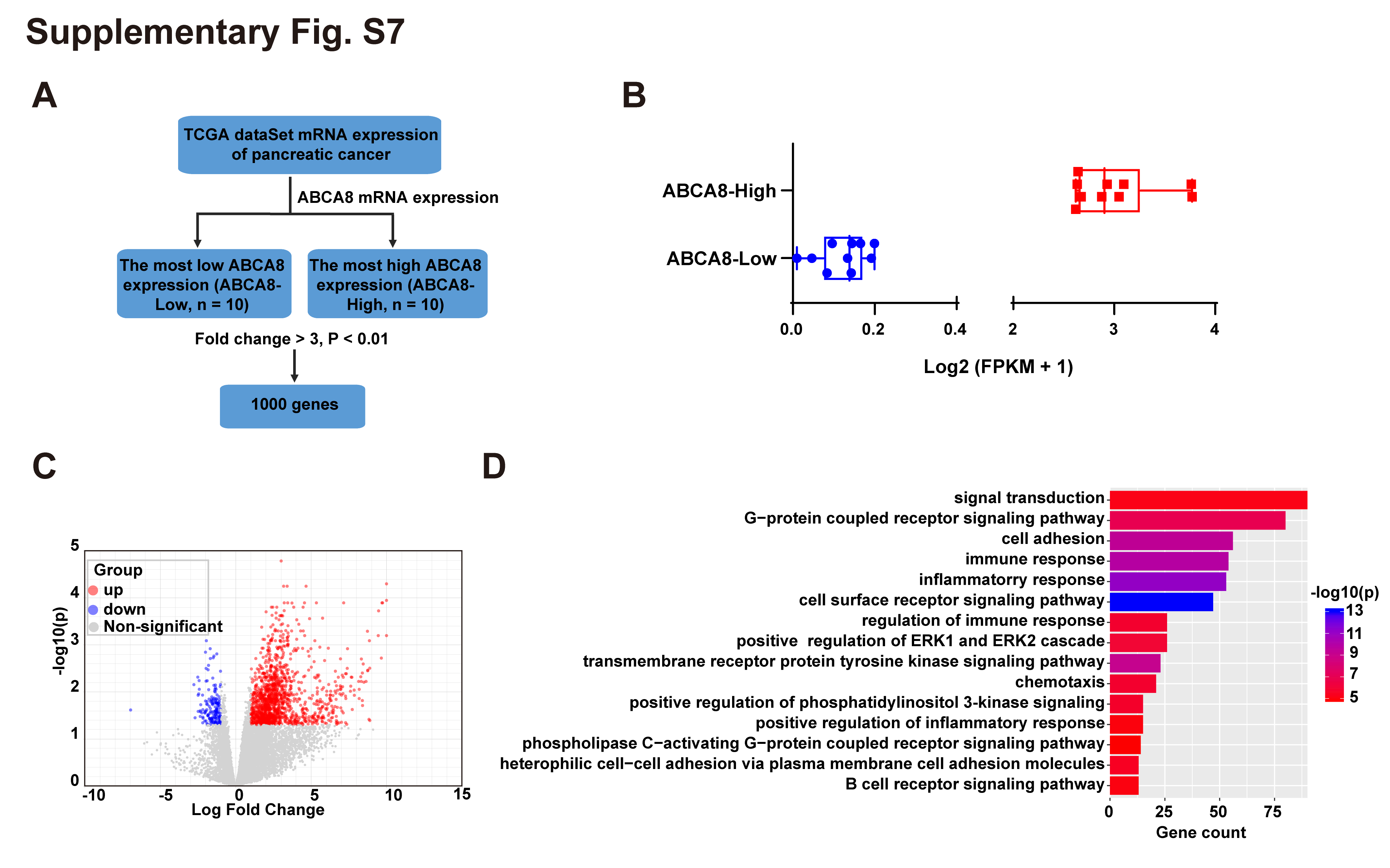

Supplement: Supplementary file 12 — Supplementary Figure S7 [file 41420_2020_390_MOESM12_ESM.tif]
